# Building a genomics-capable cancer workforce: a competency-based framework for education and training in precision oncology

**DOI:** 10.1016/j.eclinm.2026.104142

**Published:** 2026-08-10

**Authors:** Amanda Drury, Yosr Hamdi, Nicola Mulder, Jens Rueter, Lindsey Kelley, Kathleen Blazer, Raffaella Casolino, Stacy W. Gray

**Affiliations:** a School of Nursing and Midwifery, Trinity College of Dublin, Dublin, Ireland; b Trinity Centre for Practice & Healthcare Innovation, School of Nursing and Midwifery, Trinity College Dublin, Dublin, Ireland; c National Human Genomics Laboratory, Institut Pasteur de Tunis, Université Tunis El Manar, Tunis, Tunisia; d Laboratory of Biomedical Genomics and Oncogenetics (LR16IPT05), Institut Pasteur de Tunis, Université Tunis El Manar, Tunis, Tunisia; e Computational Biology Division, Institute of Infectious Disease and Molecular Medicine, Faculty of Health Sciences, University of Cape Town, Cape Town, South Africa; f The Jackson Laboratory, Bar Harbor, ME, USA; g Medical Oncology and Therapeutics Research, City of Hope, Los Angeles, CA, USA; h School of Cancer Science, University of Glasgow, Glasgow, USA; i System Strategy, City of Hope National Medical Center, Los Angeles, CA, USA

**Keywords:** Precision medicine, Genomics education, Health workforce, Competency framework, Clinical competence, Cancer

## Abstract

**Background:**

The Lancet Oncology Commission on cancer genomics and precision oncology identified workforce capability, education, and training as major constraints to equitable implementation. As genomic and multi-omic technologies become integrated into cancer care, structured approaches are needed to define competencies across the multidisciplinary workforce.

**Methods:**

The Core Competency Framework for Precision Oncology was developed using a narrative review and iterative expert-informed framework development process. Competency frameworks, professional standards, policy guidance, organisational reports, and educational literature relevant to genomic medicine, precision oncology, and workforce development were identified through purposive searches of organisational repositories, peer-reviewed literature, and reference list screening between January 2025 and March 2026. Search terms included combinations of “precision oncology”, “cancer genomics”, “genomic medicine”, “competenc∗”, “education”, “training”, and “workforce”. Competency domains, role functions, progression structures, and implementation concepts were extracted, compared, and synthesised into shared and role-specific competencies mapped across foundational, intermediate, and advanced levels. The framework was refined through iterative multidisciplinary expert consultation involving contributors to the Lancet Oncology Commission.

**Findings:**

The framework defines three components; shared core competencies applicable across professional groups; role-specific competencies aligned with functions across the precision oncology pathway; and a progression model reflecting increasing interpretive complexity, autonomy, accountability, and leadership. An associated tiered workforce model links competencies to service contexts and levels of practice, supporting distribution of genomic responsibilities while preserving escalation pathways and governance structures. The framework is designed to be adaptable across professions, health systems, and resource settings.

**Interpretation:**

This framework provides an implementation-oriented structure to support workforce development, curriculum design, continuing professional education, role mapping, competency assessment, and service planning in precision oncology. Its application may support scalable, equitable, and sustainable integration of genomics into routine cancer care.

**Funding:**

No specific funding was received for this work.

**Research in context**

**Evidence before this study**

We undertook a targeted review of competency frameworks, professional standards, policy guidance, organisational reports, and educational literature relevant to genomic medicine, precision oncology, medical education, and health workforce development. Sources were identified through purposive searches of organisational websites and repositories, peer-reviewed literature, and reference list screening. Search terms included combinations of “precision oncology”, “cancer genomics”, “genomic medicine”, “competenc∗”, “education”, “training”, and “workforce”.

Existing evidence shows that competency frameworks have been developed across disciplines, including medicine, nursing, pharmacy, and genetic counselling, alongside national and international initiatives to support genomic workforce development. However, these frameworks vary in scope, structure, and intended use, and are rarely aligned with the end-to-end implementation requirements of precision oncology across the cancer care continuum. Persistent gaps in workforce confidence, interpretation, and application of genomic testing are reported, particularly outside specialist settings.

**Added value of this study**

This work presents an implementation-oriented, cross-professional competency framework designed for precision oncology. The framework defines shared core competencies, role-specific competencies, and a progression model reflecting increasing interpretive complexity, autonomy, accountability, and leadership. It also introduces a tiered workforce model linking competencies to levels of practice and service delivery contexts. By aligning competencies with the full precision oncology pathway, the framework provides a structure to support workforce development, curriculum design, continuing professional education, role mapping, and service planning across multidisciplinary teams and diverse health-system settings.

**Implications of all the available evidence**

Workforce capability remains a major constraint to precision oncology implementation. This framework provides a practical tool to support education, workforce planning, service organisation, and governance as genomic and multi-omic technologies become integrated into routine cancer care. Implementation will require alignment between education systems, clinical workflows, governance structures, and access to specialist expertise, alongside sustained investment in workforce development. Future work should evaluate implementation, competency assessment approaches, and impacts on clinical practice, patient outcomes, and equity in access to genomic cancer care.

## Introduction

Precision oncology is transforming cancer care through the integration of genomic and multi-omic data into diagnosis, treatment selection, disease monitoring, and clinical trial matching. The Lancet Oncology Commission on cancer genomics and precision oncology highlighted both the opportunities and system-level challenges associated with this transition, emphasising that progress depends not only on scientific advances, but on the ability of health systems to implement them effectively, equitably, and sustainably.^1^ Among the Commission's key findings was that workforce capability, education, and training, together with education for patients and the public, are fundamental to this agenda.

As genomic technologies, including multigene panels, liquid biopsy, transcriptomics, proteomics, and integrated multi-omic biomarkers, become embedded in clinical guidelines, the demands placed on the cancer workforce have expanded substantially.^2^, ^3^, ^4^, ^5^ Health professionals are increasingly required to engage with complex information, interpretation, communication, and decision-making across the cancer care pathway.^6^, ^7^, ^8^, ^9^, ^10^ These responsibilities extend beyond oncology to include pathology, laboratory medicine, nursing, pharmacy, genetics, bioinformatics, clinical research, ethics, policy, and health system leadership.^8^^,^^9^^,^^11^, ^12^, ^13^

Optimal delivery of precision oncology depends on coordinated contributions across the care pathway, including biospecimen acquisition, laboratory analysis, bioinformatic interpretation, clinical reporting, treatment decision-making, communication of uncertainty, and longitudinal follow-up (Appendix 1). Yet training opportunities remain heterogeneous across professions, institutions and countries, and are often not aligned with the practical requirements of implementation.^8^^,^^12^^,^^14^^,^^15^ Gaps in workforce confidence, interpretation, and application of genomic testing have been reported, particularly as genomic testing is increasingly mainstreamed beyond specialist genetics services.^10^^,^^14^, ^15^, ^16^

Competency-based frameworks are widely used in health professional education to support curriculum design, assessment, continuing professional development, and workforce planning.^17^^,^^18^ Existing genomic competency frameworks have been developed across disciplines, including medicine, nursing, pharmacy, and genetic counselling, alongside national and international initiatives to support genomic workforce development.^18^, ^19^, ^20^, ^21^, ^22^, ^23^ However, these frameworks vary in scope, structure, and intended use, and are not consistently aligned with the end-to-end implementation requirements of precision oncology across the cancer care continuum.^24^, ^25^, ^26^, ^27^

In precision oncology, a competency-based approach can clarify which capabilities should be shared across the workforce, which should be role-specific, and how responsibility should progress as interpretive complexity and accountability increase. This paper extends the Commission's work by presenting the Core Competency Framework for Precision Oncology and its associated tiered workforce model. The purpose is to provide an implementation-oriented and adaptable framework to support curriculum development, continuing professional education, role mapping, competency assessment, service planning, and workforce policy.

## Methods

### Design

The Core Competency Framework for Precision Oncology was developed using a targeted narrative review and iterative expert-informed framework development process.^28^, ^29^, ^30^ A narrative approach was selected because the objective was to interpret and synthesise heterogeneous evidence relevant to workforce capability, competency development, and implementation of precision oncology across professional groups, rather than to aggregate homogeneous empirical studies. The evidence base includes substantial grey literature produced by professional bodies, regulators, health systems, and international organisations. Narrative reviews are appropriate where questions are broad, conceptually oriented, and require interpretive synthesis across multiple evidence types.^28^

Framework development incorporated consultation with subject matter experts in precision oncology, genomics, nursing, medical education, and workforce development to refine the structure, applicability, and role stratification of the framework.^29^^,^^30^ Consultation was undertaken to enhance interpretive validity, align the framework with contemporary clinical and workforce realities, and support applicability across professional and health-system contexts.^30^ Consistent with participatory and consultation-informed approaches, feedback was used iteratively to refine competency domains, progression levels and role expectations.^30^^,^^31^

This work formed part of a co-creative expert policy and framework development process linked to the Lancet Oncology Commission. Contributors participated as expert partners rather than research participants, and their input was reviewed and incorporated iteratively during framework and manuscript development. Formal research ethics committee approval was therefore not required. Contributors were informed of the purpose of the process, the intended use of their contributions, and the basis for acknowledgement or co-authorship.

### Search strategy and selection criteria

Consistent with methodological guidance on grey literature and purposive evidence identification, sources were identified through iterative searching with emphasis on relevance to competency development, workforce implementation, and applicability across professional and health-system contexts.^32^ Sources were identified through purposive searches of organisational websites and repositories, including those of the World Health Organization, National Human Genome Research Institute, NHS Genomics Education Programme, and professional accreditation bodies, alongside peer-reviewed publications and reference list screening. Search terms included combinations of “precision oncology”, “cancer genomics”, “genomic medicine”, “competenc∗”, “competency framework”, “education”, “training”, and “workforce”. Searches were conducted between January 2025 and March 2026. Sources were included if they described competency frameworks, curricula, professional standards, or policy guidance addressing workforce capability, education, or implementation of genomic medicine or precision oncology across professional groups (Appendix 2).^18^, ^19^, ^20^, ^21^, ^22^, ^23^, ^24^, ^25^, ^26^, ^27^^,^^33^, ^34^, ^35^, ^36^, ^37^, ^38^, ^39^, ^40^, ^41^, ^42^ Sources focused solely on laboratory technique, assay performance, or technical bioinformatics without relevance to clinical practice, workforce development, or education were excluded. Previously published Delphi-derived or consensus-based competency frameworks were eligible where they reported competency domains, role expectations, or progression structures relevant to genomic medicine or precision oncology, and were treated as source documents for narrative synthesis.

### Data synthesis and analysis

The identified material was synthesised narratively, as described in Appendix 3.^28^ Competency domains, role groupings, progression structures, and implementation-relevant concepts were extracted, compared across sources, and consolidated into shared and role-specific competency domains. Professional groups were defined according to their functions across the precision oncology pathway and mapped to International Standard Classification of Occupations (ISCO-08) categories to support global workforce policy alignment (Table 1).^43^ This functional approach was used because precision oncology roles vary across countries and service models. The framework therefore does not prescribe fixed professional boundaries; rather, it identifies competencies required for key functions that may be distributed differently depending on local regulation, workforce capacity, and service configuration.

**Table 1:** Professional groups involved in genomic testing and care pathways.

• Medical, clinical, and radiation oncologists and other medical specialists (2212) • Pathologists and laboratory medicine specialists, including molecular biologists (2212/2113) • Clinical geneticists and genetic counsellors (2212/2269) • Oncology nurses and advanced practice providers (2221/2240) • Pharmacists and clinical pharmacologists (2262/2212) • Primary care clinicians (2211) • Clinical research and trial coordination staff (2169) • Data scientists, bioinformaticians and informatics specialists (2120/2131) • Health system leaders and policy makers (1112/1342) • Bioethicists (2632), legal experts (2611/2619), social scientists (2633), and relevant policy specialists (2422)

Draft competency statements were developed across three progressive levels; foundational, intermediate, and advanced. These were mapped to knowledge, skills, and attitudes or behaviours. Draft statements were refined through iterative consultation with an international multidisciplinary expert group contributing to the Lancet Oncology Commission. Experts represented medical oncology, precision oncology, cancer genomics, bioinformatics, computational biology, artificial intelligence, pathology, radiology, molecular genetics, cancer informatics, nursing, health economics, public health, palliative care, ethics, law, governance, and health policy. The group included 58 contributors from Europe, North America, South America, Asia, Africa, and Australia, including representatives from Switzerland, Spain, the Netherlands, Italy, Colombia, Canada, the United States, the United Kingdom, Ireland, Germany, Norway, Sweden, Tunisia, South Korea, Japan, Sri Lanka, China, Nigeria, South Africa, and Australia. Feedback focused on clinical relevance, clarity, role differentiation, feasibility across settings, and alignment with implementation needs. Disagreements were resolved through iterative discussion and revision until agreement was reached. No formal Delphi process, voting procedure, or quantitative consensus threshold was used. The process was designed as an expert-informed framework development exercise, intended to synthesise existing competency evidence and support implementation, rather than to produce a formally validated consensus instrument.

During synthesis, recurring competencies appearing across multiple professional frameworks were abstracted into shared core competencies rather than assigned to a single profession. Competencies reflecting distinct responsibilities within the precision oncology pathway were organised by function rather than by fixed professional hierarchy. Progression was defined according to increasing interpretive complexity, autonomy, accountability, and governance responsibility rather than seniority alone. A separate tiered workforce model was developed to distinguish competency progression from service-level workforce configuration. The framework is intended to support adaptation and implementation across settings, but it does not prescribe curriculum content, credentialing requirements, regulatory standards, or validated assessment methods.

#### The core competency framework for precision oncology

The Core Competency Framework for Precision Oncology defines the knowledge, skills, and professional behaviours required to support safe integration of genomics and related multi-omic technologies across the cancer care continuum. It comprises shared core competencies applicable across professional groups (Table 2), role-specific competencies aligned to functions within the precision oncology pathway (Table 3), and progressive levels of competence reflecting increasing responsibility, interpretive complexity, autonomy, and leadership (Tables 4 and 5).

**Table 2:** Recommended core competencies for all professional groups.

• Basic genomic and multi-omic literacy, including genes, variants, transcriptomics, proteomics, biomarkers, somatic and germline findings. • Understanding of genomic and biomarker test purpose, indications, limitations, uncertainty, and potential implications for patients and families. • Ability to recognise clinically relevant genomic findings and potential actionability within the professionals' scope of practice. • Awareness of ethical, legal, equity, cultural, familial and psychosocial implications of genomic testing and precision oncology. • Ability to recognise when specialist referral, multidisciplinary review or escalation is required. • Commitment to maintaining competence through lifelong learning as genomic and precision oncology evidence evolves.

**Table 3:** Role-specific competencies^a^ for precision oncology by professional group (Adapted from: Accelerating equitable cancer genomics and precision oncology in health care and research: A Lancet Oncology Commission).^1^

Professional group (ISCO-08)	Scope of practice	Foundational competencies	Intermediate competencies	Advanced competencies
Medical, clinical and radiation oncologists (2212)	Clinical integration of genomic data into diagnosis, prognosis, treatment, and trial selection	Indications for tumour and germline testing; distinction between prognostic and predictive biomarkers; awareness of evidence tiers	Interpretation of complex genomic reports; integration with clinical and pathological data; communication of uncertainty	Leadership of molecular tumour boards; appraisal of complex genomic and multi-omic evidence; trial matching and off-label evidence appraisal; contribution to guideline and governance development
Pathologists and laboratory medicine specialists including molecular biologists (2212/2113)	Generation, validation, interpretation, and reporting of molecular diagnostic data	Principles of NGS, liquid biopsy and molecular diagnostic technologies; pre-analytical and analytical quality metrics	Variant classification; reporting standards; integration of molecular, histopathological and biomarker findings	Laboratory governance; validation and oversight of genomic and multi-omic assays; quality assurance; education of clinical teams
Clinical geneticists and genetic counsellors (2212/2269)	Germline risk assessment, counselling, family communication, ethical governance	Germline-somatic distinction; interpreting complex germline genomic test reports; consent; regulatory and ethical frameworks; patient education	Interpretation of germline findings; hereditary risk assessment; cascade testing; communication of results to at-risk families	Leadership in ethical governance; complex hereditary cancer assessment; policy development; integration of population screening strategies
Oncology nurses and advanced practice providers (2221/2240)	Patient education, pathway coordination, supportive genomic care	Purpose and implications of genomic testing; recognition of hereditary risk; awareness of consent and psychosocial implications	Patient education; expectation management; care coordination; and support for genomics-informed decision-making	Leadership in coordination of genomic pathways; patient navigation; communication of risk and complex results; implementation of genomics-informed care pathways
Pharmacists and clinical pharmacologists (2262/2212)	Pharmacogenomics, targeted therapy optimisation, medication safety	Pharmacogenomic principles; biomarker–drug relationships	Evaluation of pharmacogenomic and biomarker evidence for therapy selection; toxicity management and medication optimisation.	Contribution to formulary policy, institutional guidance, and multidisciplinary education
Primary care clinicians (2211)	Risk recognition, referral, survivorship and family care	Recognition of hereditary cancer risk patterns; referral pathways	Interpretation of summary genomic reports; survivorship support; coordination of long-term surveillance and referral pathways.	Integration of genomics into preventive and population health strategies
Clinical research and trial coordination staff (2169)	Omics-informed clinical research coordination; transcriptomics and proteomics data integration	Research ethics and GCP; basic cancer genomic concepts; patient consent	Coordination of genomic-driven studies; biospecimen workflows; consent process; genomic data management	Leadership in genomics research workflows and data governance
Data scientists, bioinformaticians and informatics specialists (2120/2131)	Development of clinically interpretable genomic and multi-omic systems	Understanding of clinical genomic and multi-omic contexts and data standards	Development and validation of clinically interpretable pipelines, data integration systems and decision-support tools	Governance of multi-omic integration, algorithmic transparency, AI-supported interpretation and bias mitigation
Health system leaders and policy makers (1112/1342)	System design, workforce planning, governance, equity assurance	Awareness of genomic service models and limitations	Workforce planning; service configuration; education investment and implementation; strategy development	National governance; equity frameworks; sustainability planning
Bioethicists (2632), legal experts (2611/2619), social scientists (2633), and relevant policy specialists (2422)	Ethical, legal, social, and policy analysis and governance of genomic testing and precision oncology implementation	Core ethical, legal, and social principles in genomics; awareness of data protection, consent, and equity issues	Applied ethical and legal analysis in genomic practice; policy interpretation; guidance on consent, data governance, patient rights and equitable implementation	Leadership in genomic governance and policy development; regulatory frameworks; oversight of ethical, legal, and societal implications

NGS, Next Generation Sequencing; GCP, Good Clinical Practice.

a Competencies are illustrative rather than prescriptive and should be interpreted in relation to local scope of practice, regulatory frameworks, and models of care. Progression reflects increasing interpretive complexity, autonomy, accountability, and governance responsibility rather than professional hierarchy.

**Table 4:** Progression of role-specific competencies for precision oncology across knowledge, skills, and behaviours (Adapted from: Accelerating equitable cancer genomics and precision oncology in health care and research: A Lancet Oncology Commission).^1^

Level	Knowledge	Skills	Attitudes/Behaviours
Foundational	Core genomic and biomarker concepts; purpose, indications, limits and uncertainty of testing; ethical, legal, psychosocial and equity principles	Recognise indications and referral thresholds; use basic reports within scope of practice; escalate appropriately	Openness to uncertainty; ethical awareness; respect for patient autonomy; commitment to learning
Intermediate	Evidence frameworks; interpretation principles; clinical and familial implications; role-specific applications of genomic and biomarker information	Apply genomics within defined care pathways; communicate uncertainty; support shared decision-making; coordinate care and referral	Accountability; collaboration; patient-centeredness; equity orientation
Advanced	Emerging genomic, multi-omic, liquid biopsy, AI, system and policy developments; implementation and governance frameworks	Apply transcriptomics, proteomics and other omics findings within the care pathway; lead complex interpretation or multi-disciplinary review within scope; contribute to governance, education, service design and quality improvement	Stewardship; equity-oriented leadership; systems thinking; commitment to sustainable implementation

**Table 5:** Application of competency levels across professional groups in precision oncology^a^ (Adapted from: Accelerating equitable cancer genomics and precision oncology in health care and research: A Lancet Oncology Commission).^1^

Professional group	Level	Knowledge	Skills	Attitudes/Behaviours
A1. Medical, clinical and radiation oncologists	**Foundational**	Biomarker types; tumour vs germline testing indications	Test selection; referral and escalation	Ethical responsibility
	**Intermediate**	Resistance mechanisms; VUS concepts; multi-omic biomarkers	Integrated interpretation within MDT pathways; communication of uncertainty	Shared accountability
	**Advanced**	Clinical trial evidence, multi-omic interpretation frameworks; guideline science	MTB leadership; evidence appraisal; genomics-informed treatment strategy	Stewardship of standards and governance
A2. Pathologists and laboratory medicine specialists	**Foundational**	NGS principles, liquid biopsy and molecular diagnostic principles; quality metrics	Sample, assay and biomarker assessment	Quality and precision culture
	**Intermediate**	Variant classification systems; reporting standards	Integrated molecular and histopathological reporting; Quality Assurance	Clinical accountability
	**Advanced**	Multi-omic integration and assay validation frameworks	Laboratory governance; pathway development; clinical education	Leadership in quality and translational practice
A3. Clinical geneticists and genetic counsellors	**Foundational**	Germline-somatic distinction; consent and ethical frameworks	Consent facilitation; patient and family education	Empathy and ethical awareness
	**Intermediate**	Hereditary cancer risk models; germline interpretation	Risk assessment; cascade testing; psychosocial counselling	Patient advocacy
	**Advanced**	Genomic ethics, governance and screening frameworks	Program leadership; policy development	Societal stewardship
A4. Oncology nurses and advanced practice providers	**Foundational**	Testing purpose, implications and hereditary risk concepts	Risk recognition; patient support	Patient-centred care
	**Intermediate**	Treatment implications; consent and psychosocial considerations	Education, expectation management and care coordination	Advocacy and collaborative practice
	**Advanced**	Survivorship genomics; pathway implementation principles	Genomic pathway coordination; multidisciplinary communication	Leadership in patient navigation and service implementation
A5. Pharmacists and clinical pharmacologists	**Foundational**	Biomarker–drug relationships	Therapy support	Safety focus
	**Intermediate**	Pharmacogenomic and biomarker evidence	Therapy optimisation; toxicity management	Collaborative practice
	**Advanced**	Policy and formulary science	Governance contribution	Stewardship
A6. Primary care clinicians	**Foundational**	Hereditary cancer patterns	Referral	Preventive mindset
	**Intermediate**	Genomic summaries and survivorship implications	Surveillance support; referral coordination	Continuity of care responsibility
	**Advanced**	Preventive genomics	Population integration	Public health stewardship
A7. Data scientists and bioinformaticians	**Foundational**	Clinical genomic and multi-omic contexts; data standards	Data handling and integration	Clinical awareness
	**Intermediate**	Interpretation frameworks; AI-assisted analytics	Pipeline and decision-support development	Transparency and reproducibility
	**Advanced**	AI governance; Multi-omic integration frameworks	Governance of clinically interpretable systems	Equity and algorithmic responsibility
A8. Health system leaders and policy makers	**Foundational**	Genomic service models and implementation limitations	Strategic understanding	Sustainability awareness
	**Intermediate**	Workforce, service and economic models	Investment and implementation planning	Equity orientation
	**Advanced**	National genomic policy and governance systems	Governance leadership; sustainability planning	Stewardship of equitable implementation
A9 Clinical research and trial coordination staff	**Foundational**	Research ethics; GCP; genomic testing basics	Consent processes; protocol compliance	Ethical practice
	**Intermediate**	Biomarker-driven trials; genomic datasets	Study coordination; biospecimen and genomic data management	Scientific rigour
	**Advanced**	Genomic data governance; regulatory frameworks	Research workflow leadership	Data stewardship
A10 Bioethicists, legal experts, social scientists, and relevant policy specialists	**Foundational**	Ethical and legal principles in genomics	Ethical issue recognition; consent support	Respect for autonomy; ethical awareness
	**Intermediate**	Governance frameworks; equity and societal implications	Policy interpretation; ethical guideline development	Accountability; equity orientation
	**Advanced**	Genomic policy and regulatory systems	Governance leadership; policy development	Stewardship; societal responsibility

AI, Artificial Intelligence; GCP, Good Clinical Practice; MTB, Molecular Tumour Board; NGS, Next Generation Sequencing; VUS, Variant of Uncertain Significance.

a Table 5 operationalises the competency progression described in Table 3 by illustrating how foundational, intermediate, and advanced competencies may be expressed across knowledge, skills, and attitudes/behaviours within different professional groups. The progression model was informed through synthesis of published competency frameworks, workforce guidance, and precision oncology implementation literature, and refined through multidisciplinary expert consultation. Examples are illustrative rather than prescriptive and should be interpreted in relation to local scope of practice, workforce configuration, and models of care.

The framework establishes a shared foundation for all professionals involved in cancer care, including genomic, multi-omic and biomarker literacy; understanding of test purpose, limitations and uncertainty; recognition of clinically relevant findings within scope of practice; awareness of ethical, legal, equity, familial and psychosocial implications; recognition of referral or escalation thresholds; and commitment to ongoing learning as evidence evolves (Table 2).^19^, ^20^, ^21^^,^^26^^,^^33^^,^^37^ These competencies represent minimum expectations for safe participation in genomic care as testing becomes embedded within routine oncology practice. They do not authorise independent interpretation or clinical decision-making outside professional scope but provide a baseline for recognising when genomic information is relevant and when escalation is required.

Beyond this shared baseline, the framework delineates role-specific competencies aligned with functions across the precision oncology pathway (Table 3). These include laboratory processes, clinical interpretation, treatment decision-making, patient communication, pharmacogenomic optimisation, research coordination, digital and bioinformatic interpretation, governance, and policy.^22^^,^^24^^,^^25^^,^^33^, ^34^, ^35^ The role-specific structure reflects the distributed nature of genomic care and supports alignment between scope of practice, training, supervision, and accountability. The professional groups presented in Table 3 are illustrative functional groups rather than a definitive professional taxonomy.

The three competency levels are distinguished by increasing autonomy, complexity, and accountability. Foundational competence refers to recognition, communication, appropriate referral, and safe participation within routine care.^19^, ^20^, ^21^^,^^26^^,^^33^^,^^37^ Intermediate competence refers to the application of genomic information within defined pathways, including structured risk assessment, standard report interpretation within scope, patient education, and coordination of testing or referral workflows.^21^^,^^25^, ^26^, ^27^^,^^37^ Advanced competence refers to responsibility for complex interpretation, uncertain or multi-omic findings, pathway leadership, governance, education of others, and service development.^11^^,^^18^^,^^24^^,^^34^^,^^35^^,^^39^ This progression is defined conceptually across knowledge, skills, and behaviours in Table 4, and then applied to professional groups in Table 5.

Cross-cutting capabilities, including communication, shared decision-making, management of uncertainty, ethical reasoning, equity awareness, and interpretation of complex data, apply across all levels but increase in complexity as roles expand.^18^^,^^33^, ^34^, ^35^^,^^37^^,^^40^ Their presence across the framework does not imply that accountability is diffuse or interchangeable. Shared competencies support team-based practice, but final responsibility for decisions must remain with designated professionals operating within their scope of practice, local governance structures, and escalation pathways. For example, nurses may support patient understanding and consent,^20^^,^^21^^,^^27^ pharmacists may advise on biomarker–drug relationships,^22^ and bioinformaticians may support pipeline interpretation,^11^^,^^24^ while treatment recommendations or complex variant adjudication remain the responsibility of authorised clinical or molecular specialist teams.^25^^,^^34^^,^^35^ The framework also recognises patient and public genomic literacy as part of implementation. Meaningful participation in genomic testing and precision oncology decisions requires communication strategies that enable patients and families to understand test purpose, limitations, uncertainty, potential hereditary implications, and available options for action.^23^^,^^33^

#### Applying the framework in practice

The framework is intended for workforce planners, educators, curriculum developers, professional societies, accreditation bodies, healthcare organisations, cancer centres, clinical leaders, and policymakers seeking to operationalise education and training in precision oncology. Although relevant to clinicians and other healthcare professionals, its primary purpose is not to provide a point-of-care resource for individual clinicians, but to support workforce planning, curriculum development, professional standards, accreditation processes, and capacity-building initiatives in cancer genomics and precision oncology. It is not designed as a stand-alone curriculum, assessment tool, credentialing standard, or regulatory instrument. Instead, it provides an adaptable structure to support curriculum design, continuing professional development, role mapping, service and governance planning, and future competency assessment. Professional groups included in the framework represent key functions across the precision oncology pathway rather than a definitive taxonomy. Additional groups, including psychologists, allied health professionals, and other specialist roles, may be incorporated through local adaptation where they contribute to genomic testing, interpretation, communication, care coordination, governance, or implementation.

The framework is intended to support workforce planning across health systems at different stages of precision oncology development, from settings beginning to establish cancer genomics capacity to those with advanced genomic and multi-omic services. It should be viewed as a flexible roadmap for workforce development rather than a prescriptive model tied to a particular health system. Competencies may be prioritised, adapted, and implemented incrementally according to local needs, resources, and stages of health-system maturity. In established systems, it may support service refinement, molecular tumour board functioning, accreditation processes, credentialing, and continuing professional development. In emerging or resource-constrained systems, it may support phased workforce development, minimum safe competencies, hub-and-spoke models, and task-sharing aligned with available diagnostics and treatments.

Education providers may use the framework to map curricula against expected competencies; cancer centres to identify training gaps; professional bodies to inform standards; and health systems to align workforce capability with precision oncology service models. Across the education continuum, foundational competencies can be embedded in undergraduate and pre-registration education to establish baseline genomic literacy, while postgraduate education can support role-specific competencies aligned with professional scope and levels of responsibility. For the existing workforce, targeted continuing professional development remains important given persistent gaps in genomic confidence, interpretation, and application across settings.^10^^,^^15^^,^^44^ Short modular formats, micro-credentials, case-based learning, and workplace-based supervision may be more feasible than extended programmes for clinicians working in time-constrained services.

Formal education alone is insufficient. Competency development also requires practice-embedded learning models, including supervised clinical exposure, mentorship, multidisciplinary case discussion, and point-of-care decision support (Table 6).^12^ Molecular tumour boards are important because they function as both decision-making structures and learning environments, exposing clinicians to real-world interpretation, uncertainty, evidence appraisal, and multidisciplinary accountability.^34^^,^^35^^,^^52^ Digital clinical decision-support tools can reinforce learning within routine workflows, but should supplement rather than replace professional judgement, supervision, and governance.^50^^,^^51^

**Table 6:** Education and training models supporting precision oncology implementation.

Model	Description	Examples
Scientific training programs	Didactic classroom-based learning designed to establish competency or mastery in specific areas such as genetics or bioinformatics	Undergraduate, pre-curricular, Masters or PhD programs
Clinical training programs	Formal clinical training designed to establish competency or mastery in specific areas such as genetic counselling, molecular pathology, or medical oncology	Master's degree; Residency, or fellowship programs
Intensive training programs	Comprehensive certificate training covering germline genetics and topics in precision oncology	COH Intensive Course and Community of Practice^45^^,^^46^
Short courses, workshops	Short-format training (one to multiple days) that covers specific topics in precision oncology	Pre-conference workshops, continuing professional development courses.
Multidisciplinary case-based learning	Interprofessional collaborative forums designed for hands-on data analyses in the context of patient case presentations	Molecular tumour boards; Graduate school courses^47^
Communication skills training	Programs designed to aid providers in communicating complex genomic data with patients	Structured communication skills training^48^
E-Learning	Web-based training courses designed to address specific topics in precision oncology	e-Learning courses in oncogenomics, JAX Clinical Education^9^^,^^49^
Point of care education & decision support	Informatics-based strategies designed to educate providers in real time at the point of care as they navigate clinical questions. These tools are frequently, but not uniformly, integrated into electronic health record systems	EPIC Genomics, CDS^50^^,^^51^
Workplace-based clinical training	Structured training delivered within routine clinical services to support role expansion and competency development in precision oncology.	In-service training; mentorship within mainstreaming pathways; supervised practice; competency validation; pathway-embedded training^12^

COH, City of Hope; JAX, Jackson Laboratory; CDS, Clinical Decision Support.

At service level, the framework can support role mapping and task distribution by identifying competencies required for different levels of responsibility, interpretive complexity, and service delivery within precision oncology pathways. This is particularly relevant in mainstreamed models of genomic care, where testing is increasingly delivered outside specialist genetics services. The Tiered Workforce Model for Precision Oncology (Table 7) operationalises this structure across three levels of practice; precision-informed, enhanced, and specialist. At Level 1, professionals providing routine cancer services require basic genomic literacy, recognition of hereditary or genomic risk indicators, communication of test purpose and limitations, and appropriate referral or escalation. At Level 2, professionals with additional training apply genomic information within defined pathways, including structured risk assessment, consent, standard report interpretation within scope, care coordination, and referral. At Level 3, professionals whose primary role includes genomics or precision oncology undertake complex interpretation, lead molecular tumour boards or expert consultation, oversee pathways, and contribute to governance, service development, and policy.

**Table 7:** Tiered workforce model for precision oncology: levels of practice, context, core activities and competencies.^a^

Level of practice	Service context	Professional practice context	Core activities and competencies
Level 3: Specialist precision cancer care practice	Specialist genomic services providing complex interpretation, oversight, and system leadership	Delivered by professionals whose primary role includes genomics and precision oncology.	Interpretation of complex genomic and multi-omic findings, including variants of uncertain significance and integrated biomarker data; leadership of molecular tumour boards and expert consultation; oversight of genomic testing pathways, governance and quality assurance; management of complex hereditary risk and cascade testing; service development, evaluation, and policy leadership.
Level 2: Enhanced precision cancer care	Mainstream oncology services delivering defined genomic testing or interpretation pathways	Delivered by professionals within mainstream oncology services who have additional genomics training.	Structured genomic risk assessment and pedigree documentation; provision of pre-test education and consent within defined clinical pathways; application and interpretation of standard genomic reports within scope of practice; coordination of genomic testing workflows and referral pathways; management of common germline and somatic findings within established escalation pathways.
Level 1: Precision-informed cancer care	Routine cancer services incorporating basic genomic awareness and referral pathways	Delivered by all professionals providing routine cancer services.	Basic genomic literacy; recognition of hereditary cancer risk indicators; understanding the purpose, limitations, and uncertainty of genomic testing; communication of genomic information, limitations and uncertainty with patients; recognition of when referral or escalation is required.

a The tiered workforce model is intended to support adaptation across different workforce configurations and health-system contexts. Levels reflect increasing complexity, autonomy, accountability, and governance responsibility rather than professional hierarchy.

Table 8 illustrates how responsibilities may be distributed across professional groups within this tiered model. The examples are illustrative rather than prescriptive and show that progression reflects interpretive complexity, autonomy, accountability, and governance responsibility rather than professional hierarchy. Competency assessment is essential if the framework is to function as an implementation tool. Assessment approaches should be locally adapted, but may include self-assessment tools to identify learning needs; knowledge tests for foundational concepts; case-based assessment of report interpretation and referral decisions; observed structured clinical encounters for consent and communication; portfolio evidence of supervised practice; educational evaluations of training programmes; audit of appropriate test use and referral patterns; participation in molecular tumour board review; and, where appropriate, certification or credentialing processes developed by professional, educational, or regulatory bodies. Advanced competence may additionally require evidence of leadership, governance contribution, quality improvement, education of others, or service development. These approaches would allow organisations to link competency expectations to training, supervision, credentialing, and quality assurance.

**Table 8:** Operationalisation of the tiered workforce model for precision oncology across professional groups.

Professional group	Level 1: Precision-informed cancer care	Level 2: Enhanced precision oncology practice	Level 3: Specialist precision oncology practice
*Service context*	*Routine cancer services incorporating basic genomic awareness and referral pathways*	*Mainstream oncology services delivering defined genomic testing or interpretation pathways*	*Specialist genomic services providing complex interpretation, oversight, and system leadership*
Medical, clinical and radiation oncologists (2212)	Recognise indications for genomic testing; discuss purpose and limitations; refer appropriately	Interpret standard genomic reports; integrate findings into treatment decisions; communicate uncertainty	Lead complex interpretation; chair molecular tumour boards; guide trial matching and off-label use
Pathologists and laboratory medicine specialists including molecular biologists (2212/2113)	Ensure appropriate sample handling; understand testing workflows	Interpret and report validated genomic findings; integrate molecular and histopathological data	Lead assay development and validation; oversee multi-omic integration and laboratory governance
Clinical geneticists and genetic counsellors (2212/2269)	Support referral pathways; recognise hereditary risk triggers	Conduct risk assessment; provide pre- and post-test counselling; coordinate cascade testing	Lead complex counselling, ethical governance, and population-level genomic strategies
Oncology nurses and advanced practice providers (2221/2240)	Recognise hereditary risk; explain purpose and limits of testing; support referral	Deliver genomic education; support consent and shared decision-making; coordinate care pathways	Lead genomic navigation; communicate complex results and risk; contribute to pathway development and service improvement
Pharmacists and clinical pharmacologists (2262/2212)	Recognise biomarker–drug relationships; support safe prescribing	Evaluate genomic evidence for therapy selection; manage toxicity and drug interactions	Contribute to formulary decisions; develop institutional guidance and education
Primary care clinicians (2211)	Recognise hereditary cancer patterns; initiate referral	Support long-term follow-up and surveillance; interpret summary reports	Integrate genomics into prevention and population health strategies
Clinical research and trial coordination staff (2169)	Support consent and protocol adherence in genomic studies	Coordinate biomarker-driven trials; manage genomic datasets	Lead genomic research workflows; contribute to data governance and translational research
Data scientists, bioinformaticians and informatics specialists (2120/2131)	Understand clinical context of genomic data	Develop and apply analysis pipelines; support clinical interpretation tools	Lead multi-omic integration; oversee algorithm development and governance
Health system leaders and policy makers (1112/1342)	Understand genomic service models and workforce needs	Plan workforce development and education strategies; support implementation	Lead policy, governance, and national genomic strategy
Bioethicists (2632), legal experts (2611/2619), social scientists (2633), and relevant policy specialists (2422)	Recognise ethical, legal, and social issues in genomic care; raise awareness	Apply ethical and legal frameworks; support consent, data use, and equity in practice	Lead governance, policy development, and oversight of ethical, legal, and societal implications

Roles are illustrative and may vary depending on workforce configuration, regulation, and resource context.

#### Implementation considerations across settings

Implementation will require alignment between workforce capability, service configuration, diagnostic infrastructure, treatment availability, and governance. The framework cannot create sequencing capacity, reimbursement mechanisms, bioinformatics infrastructure, access to targeted therapies, or specialist workforce supply. Its value lies in helping health systems define competencies required for safe practice, identify workforce gaps, support task-sharing, and establish escalation pathways appropriate to local resources and models of care.

Implementation priorities will differ across settings. In specialist centres, emphasis may be placed on advanced interpretation, multi-omic integration, molecular tumour board quality, computational oncology, and institutional credentialing. In non-specialist settings, priorities may include foundational genomic competence across the workforce, defined pathways for test ordering and referral, and access to regional specialist consultation. Across both contexts, clear escalation thresholds remain essential for uncertain findings, potential germline implications, rare variants, complex resistance mechanisms, multi-omic signatures, and off-label treatment considerations.^34^^,^^35^

Implementation in low-income and middle-income countries and other resource-constrained settings require caution. Workforce shortages, limited pathology capacity, constrained access to sequencing, variable reimbursement, and limited availability of matched therapies may restrict what precision oncology services can deliver.^3^^,^^53^^,^^54^ In these contexts, phased implementation may be more appropriate than comprehensive immediate adoption. Early priorities may include foundational genomic literacy, recognition of hereditary risk, appropriate referral pathways, specimen quality, informed consent, and avoidance of low-value or non-actionable testing.^21^^,^^26^ Intermediate and advanced competencies may then be concentrated in designated hubs, regional networks, or centres of excellence linked to virtual molecular tumour boards and consultation pathways.^34^^,^^35^

Equity requires that competency development be aligned with locally actionable care. Genomic testing without capacity for interpretation, counselling, referral, treatment, surveillance, or follow-up can create avoidable costs, delays, uncertainty, and inequitable access to benefit. Workforce development should therefore be linked to available diagnostics, treatment pathways, trial access, family risk services, and palliative or supportive care. This alignment may help prevent precision oncology from remaining concentrated in specialist centres or becoming aspirational rather than clinically actionable.

Increasing integration of multi-omic and liquid biopsy technologies also creates implementation and workforce requirements. As transcriptomic, proteomic, epigenomic, immune profiling, and longitudinal circulating tumour DNA approaches enter clinical practice, health professionals require competencies in assay selection, specimen acquisition and timing, pre-analytical and analytical limitations, longitudinal interpretation, resistance monitoring, and integration of multiple molecular and clinical data streams.^11^^,^^24^ These developments increase demand for advanced capabilities in molecular pathology, bioinformatics, computational analysis, and clinical interpretation, alongside the ability to communicate probabilistic findings, uncertainty, and evolving evidence to patients and multidisciplinary teams.^24^

AI and computational oncology introduce further competency requirements as precision medicine increasingly depends on biomedical informatics, omics data integration, clinical decision support, and data governance.^24^ Genomic clinical decision-support systems and electronic health record-based genomic modules may improve the integration of pharmacogenomic and genomic information into clinical workflows, but require structured data, validated interpretation logic, multidisciplinary implementation, maintenance, and attention to clinician- and patient-facing communication.^50^^,^^51^ AI-assisted interpretation, automated trial matching, and multi-omic prediction tools raise additional requirements relating to transparency, validation, explainability, bias, data governance, and over-reliance on automated outputs.^24^ Competence in precision oncology therefore includes the ability to critically appraise digital tools, recognise when outputs require expert multidisciplinary review, and ensure that algorithmic outputs are interpreted within clinical evidence, equity considerations, and accountable professional judgement.^24^^,^^50^^,^^51^ As these technologies mature, the framework will require periodic review to ensure that competency expectations remain aligned with clinical practice, governance requirements, and workforce roles.

## Discussion

The Core Competency Framework for Precision Oncology responds to priorities identified by the Lancet Oncology Commission by translating broad workforce and education recommendations into an implementation-oriented structure for competency development across multidisciplinary cancer care.^1^ Existing genomic competency initiatives have largely been profession-specific or focused on discrete components of genomic medicine. By integrating shared competencies, role-specific expectations, progression pathways, and workforce stratification within a single framework, this work provides a more operational approach to supporting precision oncology implementation across diverse health-system contexts.

The literature increasingly characterises genomic competence as distributed across multidisciplinary teams rather than confined to specialist genetics services, particularly as precision oncology workflows expand from single biomarkers toward multi-gene panels, liquid biopsy, and integrated multi-omic approaches.^11^^,^^55^ Precision oncology involves an end-to-end interpretive process spanning test selection, specimen handling, assay interpretation, treatment decision-making, patient communication, longitudinal follow-up, and governance.^6^^,^^9^ As these responsibilities become distributed, the interpretive and coordination burden increases across professional groups, reinforcing the need for competency thresholds, escalation pathways, and multidisciplinary accountability.

Mainstreaming genomic testing outside specialist genetics services creates tension between expanding access and maintaining interpretive quality, governance, and patient safety.^55^ Previous studies have identified risks associated with insufficient genomic training and support, including inappropriate test ordering, misinterpretation, delayed referral, and missed opportunities for treatment or familial risk management.^14^^,^^16^ These challenges highlight the limitations of workforce models that rely primarily on professional boundaries without defining competency expectations across levels of practice. The tiered workforce model proposed here addresses this issue by aligning genomic responsibilities with competency thresholds, supervision requirements, and escalation pathways, while allowing responsibilities to be distributed flexibly across multidisciplinary teams and health-system contexts.

Existing evidence also suggests that genomic education cannot be separated from service delivery models and clinical workflow integration. Didactic teaching may establish foundational knowledge, but competence in precision oncology requires application within complex, uncertain, and multidisciplinary clinical environments.^14^ Practice-embedded learning models, including supervised clinical exposure, molecular tumour boards, mentorship, multidisciplinary case review, and clinical decision-support tools, are therefore likely to be central to sustainable implementation.^12^^,^^34^^,^^35^^,^^52^ These approaches allow competency development through exposure to real-world interpretation, evidence appraisal, communication of uncertainty, and shared accountability.

The benefits of precision oncology depend not only on access to testing, but on workforce capacity to support appropriate test selection, interpretation, communication, referral, and access to actionable pathways. Where workforce capability, interpretive support, or treatment access are limited, genomic testing may fail to translate into clinical benefit and may increase delays, costs, uncertainty, or inequities. This is particularly relevant in low-income and middle-income countries and other resource-constrained settings, where genomic services may emerge through isolated centres or research collaborations without broader workforce, governance, or infrastructure capacity.^53^^,^^54^ In such settings, phased implementation, hub-and-spoke service models, virtual molecular tumour boards, and minimum safe competency thresholds may help extend access while maintaining quality and escalation pathways.

At system level, implementation will require integration of competencies into education, credentialing, workforce planning, governance, and quality assurance structures. Both underuse and misuse of genomic testing have been associated with clinician knowledge and confidence gaps.^10^^,^^14^ If competency frameworks are to support implementation rather than function as reference documents, they should be linked to measurable indicators such as appropriate test selection, referral quality, consent documentation, report interpretation, molecular tumour board participation, patient understanding, time to actionable treatment, and equity of access. Until such assessment and implementation strategies are developed and tested, the framework should be regarded as a foundational resource for workforce development rather than a validated assessment or credentialing instrument. With integration into training, supervision, and quality systems, competency frameworks may function as practical implementation tools.

Several areas require further development and validation. Further work should focus on piloting the framework within educational and training programmes and evaluating its impact on curriculum development, workforce capability, and professional practice. Evaluation across different professional, regulatory, and resource settings would help determine feasibility, applicability, and adaptation needs. Until such implementation and evaluation have been undertaken, the framework should be regarded as a foundational resource for workforce development rather than a validated assessment or credentialing instrument.

### Limitations

Several limitations should be acknowledged. The framework was developed through an iterative expert-informed framework development process and has not undergone external validation or prospective evaluation. The professional groupings within the framework are simplified to support global applicability and may not capture all relevant roles, hybrid positions, or country-specific scopes of practice. The framework does not prescribe curriculum content, training duration, assessment tools, credentialing standards, or regulatory requirements; these must be developed within local educational and governance systems. Although the framework is intended to support multi-omic precision oncology, many existing competency sources remain genomics-focused. Periodic refinement will be needed as transcriptomic, proteomic, liquid biopsy, AI-assisted, and computational oncology applications mature to ensure alignment with technological, clinical, and workforce developments. Because the framework was developed through interpretive synthesis, some domains, particularly those relating to multi-omic integration, liquid biopsy, AI-assisted interpretation, computational oncology, and workforce stratification, were constructed by integrating source concepts with expert-informed judgement rather than being directly reproduced from existing competency frameworks. Finally, implementation in resource-constrained settings will depend on infrastructure, specialist support, treatment access, and policy investment beyond the scope of the framework itself.

### Conclusion

The Core Competency Framework for Precision Oncology provides a structured approach to operationalising workforce, education, and training priorities identified by the Lancet Oncology Commission. By defining shared and role-specific competencies, distinguishing levels of responsibility, and linking competencies to service delivery, it provides a foundation for curriculum development, continuing professional education, role mapping, competency assessment, service planning, and workforce development. The framework is not intended as a stand-alone curriculum, assessment tool, credentialing standard, or regulatory instrument. It is an adaptable structure that can be applied across health-system contexts to support the development of a genomics-capable cancer workforce and the scalable, equitable, and sustainable integration of precision oncology into routine care.

## Contributors

RC lead the concept and design of the work. RC developed the methodological approach, with input from AD, SWG, YH, NM, JR, LK, and KB. AD, YH, NM, JR, LK, KB, RC, and SWG contributed to the identification, synthesis, interpretation, and curation of source material informing the framework. All authors accessed and verified the data. AD prepared the first draft of the manuscript, with input from RC and SWG. All authors contributed to the interpretation of the framework, critically reviewed the manuscript, approved the final version for submission, and agreed to be accountable for the work.

## Data sharing statement

The datasets generated and analysed during the current study are not publicly available. Excerpts of data are available upon reasonable request.

## Declaration of interests

JR reports grants or contracts from the Harold Alfond Foundation and Hope Foundation in the past 36 months. KRB reports grants or contracts from the National Cancer Institute R25 programme, AstraZeneca Medical Education in Global Oncology, and the US Department of Veterans Affairs in the past 36 months; payments were made to her institution. AD, YH, NM, LK, RC, and SWG declare no competing interests.
