# Supplementary material for: Building a genomics-capable cancer workforce: a competency-based framework for education and training in precision oncology
Source: eClinicalMedicine. 2026 Aug 10;99:104142. doi: 10.1016/j.eclinm.2026.104142 (PMC13486856; doi:10.1016/j.eclinm.2026.104142)
Supplement: Supplementary file 1 [file mmc1.docx]

**Supplementary Material**

Drury, A., Hamdi, Y., Mulder, N., Reuter, J., Kelley, L., Blazer, K., Casolino, R., Gray, S.W. (2026) Building a genomics-capable cancer workforce: a competency-based framework for education and training in precision oncology. eClinicalMedicine.

Contents

[Appendix 1: Multidisciplinary professional groups contributing across the precision oncology pathway	2](#_Toc235727282)

[Appendix 2: Competency frameworks informing development of the Core Competency Framework for Precision Oncology	3](#_Toc235727283)

[Appendix 3: Development and synthesis of the core competency framework and tiered workforce model for precision oncology	4](#_Toc235727284)

**Appendix 1: Multidisciplinary professional groups contributing across the precision oncology pathway**

# Appendix 2: Competency frameworks informing development of the Core Competency Framework for Precision Oncology

1. A cross-professional competency framework for communicating genomic results.^33^
2. Accreditation Council for Genetic Counseling Practice-Based Competencies for Genetic Counselors.^23^
3. American Nurses Association Essentials of genomic nursing: Competencies and outcome indicators.^20^
4. CAN.HEAL consortium recommendations for Molecular Tumor Boards.^34^
5. Competencies in Cancer Genomics for Healthcare Professionals.^25^
6. ESMO Precision Oncology Working Group recommendations on the structure and quality indicators for molecular tumour boards in clinical practice.^35^
7. Essential Genetic and Genomic Competencies.^36^
8. Framework for development of physician competencies in genomic medicine: report of the Competencies Working Group of the Inter-Society Coordinating Committee for Physician Education in Genomics.^19^
9. NHS England Facilitating genomic testing: A competency framework.^26^
10. NHS England The 2023 Genomic Competency Framework for UK Nurses.^27^
11. NHS England Communicating germline genomic results: A competency framework.^37^
12. Personalized Precision Medicine for Health Care Professionals: Development of a Competency Framework.^24^
13. Pharmacists Leading the Way to Precision Medicine: Updates to the Core Pharmacist Competencies in Genomics.^22^
14. Physician Assistant Genomic Competencies.^38^
15. Update to the essential genomic nursing competencies and outcome indicators.^21^
16. World Health Organization Enhanced global competency model.^39^
17. World Health Organization Global competency and outcomes framework for the essential public health functions.^40^
18. World Health Organization Global competency framework for universal health coverage.^18^
19. World Health Organization Rehabilitation Competency Framework.^41^
20. World Health Organization – Association of Schools of Public Health in the European Region (WHO-ASPHER) Competency Framework for the Public Health Workforce in the European Region.^42^

**Appendix 3: Development and synthesis of the core competency framework and tiered workforce model for precision oncology**

The shared core competencies across professional groups (Table 2), role-specific competencies by professional group (Table 3; Table 5), progression across foundational, intermediate, and advanced levels of competence (Table 4), and tiered workforce model for precision oncology practice (Table 7) were developed through iterative synthesis of established genomic, professional, educational, implementation, and workforce competency frameworks, followed by expert consultation and refinement. The objective was to identify competencies relevant to safe and effective precision oncology implementation across professional groups and levels of practice, and to operationalise these into an implementation-oriented competency framework and workforce model.

The source documents included cross-professional genomic communication frameworks,^33^ profession-specific competencies for nurses,^20,21^ physician assistants,^38^ pharmacists,^22^ and genetic counsellors,^23^ alongside broader precision medicine, molecular tumour board, implementation, and workforce frameworks.^18,24,34,35,39,40^ Competency domains were extracted and compared across sources, then grouped according to recurring role functions, including genomic test selection, laboratory analysis, interpretation of genomic findings, communication, consent, psychosocial support, pharmacogenomic optimisation, care coordination, governance, and implementation. Table S1 summarises the main competency domains identified across the reviewed literature and the refinements introduced through expert consultation.

During synthesis, competencies recurring across multiple professional and educational frameworks were abstracted into shared core competencies. These included genomic literacy, understanding of test purpose and limitations, management of uncertainty, ethical and psychosocial awareness, recognition of referral or escalation thresholds, collaboration, and lifelong learning. These domains informed the core competency framework (Table 2). NHS England genomic testing and communication frameworks were particularly relevant to scope-specific responsibilities, consent, uncertainty, and escalation pathways,^26,27,37^ while molecular tumour board and implementation guidance informed the need for specialist multidisciplinary interpretation and escalation for complex findings.^34,35^

Competencies linked to specific functions within the precision oncology pathway were mapped as role-specific rather than assigned according to professional hierarchy. This functional approach informed Table 3 and Table 5. Oncology competencies were derived from literature emphasising biomarker interpretation, evidence appraisal, treatment integration, and communication of uncertainty.^25,35^ Pathology and laboratory medicine competencies reflected molecular diagnostics, quality assurance, variant interpretation, and multi-omic integration.^11,24^ Nursing competencies focused on patient education, supportive genomic care, pathway coordination, advocacy, and genomics-informed communication.^20,21^ Pharmacist competencies reflected pharmacogenomics and medication optimisation,^22^ while primary care competencies reflected hereditary cancer risk recognition, referral, survivorship, and preventive genomics.^19,38^ Data science and bioinformatics competencies reflected clinically interpretable pipelines, multi-omic systems, and AI-supported interpretation.^11,24^

Competency progression was defined by increasing interpretive complexity, contextual application, autonomy, accountability, leadership, and governance responsibility rather than by professional seniority alone. This decision was informed by workforce and genomic competency frameworks that distinguish broad participation in genomic pathways from specialist interpretation, leadership, and governance responsibilities.^26,39^ Foundational competencies were therefore defined as recognition, participation, communication, and referral within defined pathways; intermediate competencies as application of genomic information within professional scope and established care pathways; and advanced competencies as complex interpretation, governance, leadership, education, and service development. This progression informed Table 4 and its professional group applications in Table 5.

A separate tiered workforce model was developed to distinguish individual competency progression from service-level workforce configuration. Literature on mainstreamed genomic care, workforce organisation, molecular tumour boards, and implementation pathways distinguished between broad workforce genomic awareness and specialist interpretive expertise.^34,35,50^ Table 7 therefore conceptualised precision oncology practice across precision-informed, enhanced, and specialist levels, reflecting increasing complexity, autonomy, accountability, and governance responsibility across service contexts while preserving escalation pathways and access to specialist expertise.

The initial competency framework and workforce model were refined through consultation with the multidisciplinary international expert group contributing to the Lancet Oncology Commission. Consultation refined role boundaries, competency progression, escalation pathways, and distinctions between foundational, intermediate, advanced, and specialist practice. It also strengthened explicit incorporation of equity, governance, multidisciplinary accountability, multi-omic interpretation, AI-supported interpretation, and implementation across diverse health system contexts. Expert consultation was particularly important for rapidly evolving areas that were less consistently represented in existing competency frameworks, including multi-omic integration, liquid biopsy workflows, computational oncology, AI-supported interpretation, algorithmic governance, and implementation in resource-constrained settings. In these areas, expert feedback was used to refine language and align the framework with contemporary precision oncology practice while acknowledging the need for future validation.

***Table S1. Mapping of review findings and external competency frameworks to the proposed competency domains***

| Competency Domain | Key Findings from Included Literature and Frameworks | Refinement Through Expert Consultation Process |
| --- | --- | --- |
| Genomic literacy and biomarker knowledge | Frameworks described baseline understanding of somatic and germline genomics, biomarkers, sequencing technologies, genomic testing pathways, and the relationship of genomics to prevention, diagnosis, prognosis, treatment selection, and monitoring as relevant across professional groups.^19-21,25,26,38^ | Expanded to include multi-omic concepts, liquid biopsy technologies, and biomarker interpretation language relevant to contemporary precision oncology implementation. |
| Interpretation and contextual application of genomic findings | Sources identified increasing requirements for interpretation of genomic reports, integration with clinical and pathological context, evidence appraisal, actionability assessment, communication of uncertainty, and recognition of when complex findings require specialist or molecular tumour board review.^24,25,34,35,37^ | Clarified progression from foundational recognition and referral to intermediate application within defined pathways, to advanced interpretation, molecular tumour board leadership, and governance responsibilities. |
| Consent, communication, shared decision-making, and psychosocial support | Frameworks described informed consent, patient education, family communication, psychosocial support, health literacy, shared decision-making, and communication of uncertain or familial implications as important components of genomic care.^20,21,23,33,37^ | Expanded to include expectation management, communication of uncertainty, genomics-informed decision support, and patient/family-facing communication across professional groups. |
| Referral, escalation, and scope of practice | Frameworks differentiated between foundational participation in genomic pathways and specialist interpretation or management responsibilities, including recognising professional boundaries, seeking assistance, and referring to specialist genomic services when appropriate.^19-21,26,27,33^ | Incorporated explicit language relating to referral pathways, escalation thresholds, multidisciplinary review, and interpretation within scope of practice. |
| Multidisciplinary collaboration and pathway coordination | Precision oncology literature described genomic care as a multidisciplinary process involving coordination across laboratory, clinical, nursing, pharmacy, research, bioinformatics, ethics, policy, and governance functions.^20,24,26,34,35^ | Expanded competencies relating to pathway coordination, multidisciplinary accountability, role clarity, and integration across the precision oncology care pathway. |
| Pharmacogenomics and treatment optimisation | Pharmacogenomic and precision medicine frameworks described competencies relating to biomarker-drug relationships, targeted therapy selection, medication safety, toxicity management, drug-gene interactions, and medication optimisation.^22,24,25^ | Refined into role-specific competencies for pharmacists, clinical pharmacologists, oncology professionals, and other professionals involved in targeted therapy selection, toxicity management, and medication safety. |
| Data science, bioinformatics, clinical decision support, and AI-supported interpretation | Literature described increasing use of clinically interpretable pipelines, biomedical informatics, genomic and multi-omic data integration, clinical decision-support systems, and computational approaches in precision oncology.^11,22,24^ | Expanded to include algorithmic transparency, AI-supported interpretation, explainability, bias mitigation, data governance, and the need for accountable professional judgement when using computational outputs. |
| Multi-omic and liquid biopsy workflows | Precision oncology sources described increasing integration of liquid biopsy, longitudinal molecular monitoring, assay selection, specimen timing, resistance monitoring, and integration of multiple molecular data streams into clinical decision-making.^11,24,34,35^ | Added explicit multi-omic and liquid biopsy language to avoid a genomics-only framework and to reflect contemporary precision oncology workflows. Expert consultation helped avoid over-specification in a rapidly evolving area. |
| Governance, implementation, equity, and sustainability | Workforce, public health, and policy frameworks described governance, workforce planning, implementation capability, sustainability, quality assurance, health-system alignment, and equitable access as necessary components of genomic service delivery.^18,24,39-41^ | Strengthened explicit incorporation of governance, equity, sustainability, implementation readiness, and health-system adaptation across competency levels and professional groups. |
| Molecular tumour board governance and specialist multidisciplinary interpretation | Molecular tumour board literature described specialist multidisciplinary review, evidence appraisal, interpretation of complex or uncertain findings, quality indicators, referral pathways, and governance processes as central to precision oncology implementation.^34,35^ | Incorporated molecular tumour boards as both governance structures and practice-embedded learning environments, particularly at advanced/specialist levels of practice. |
| Competency progression and workforce stratification | Workforce competency frameworks described progression according to increasing autonomy, complexity, accountability, leadership, and governance responsibility, while genomic frameworks distinguished foundational participation from specialist interpretation or management.^18,26,27,33,39^ | Applied a progression model across all professional groups to distinguish foundational, intermediate, and advanced competencies without implying professional hierarchy. Expert consultation also clarified the distinction between competency levels and service/workforce tiers. |
| Education, competency assessment, and practice-embedded learning | Educational and competency frameworks described the need for curriculum mapping, continuing professional development, competency assessment, reflective practice, direct observation, case-based learning, workplace learning, and maintenance of competence.^18,20,21,27,40^ | Expanded to include practice-embedded learning, supervised clinical exposure, molecular tumour board participation, competency assessment, portfolios, case-based assessment, and ongoing professional development as implementation requirements rather than optional educational add-ons. |
